# Incidence of new onset type 2 diabetes in adults living with obesity treated with tirzepatide or semaglutide: real world evidence from an international retrospective cohort study

**DOI:** 10.1016/j.eclinm.2024.102777

**Published:** 2024-08-15

**Authors:** Matthew Anson, Alex E. Henney, Nicholas Broadwell, Sizheng S. Zhao, Gema H. Ibarburu, Gregory Y.H. Lip, John P.H. Wilding, Daniel J. Cuthbertson, Uazman Alam

**Affiliations:** a Diabetes & Endocrinology Research, Institute of Life Course and Medical Sciences, University of Liverpool and Liverpool University Hospital NHS Foundation Trust, Liverpool, UK; b Manx Care, Isle of Man, UK; c Centre for Musculoskeletal Research, Division of Musculoskeletal and Dermatological Science, School of Biological Sciences, Faculty of Biological Medicine and Health, The University of Manchester, Manchester Academic Health Science Centre, Manchester, UK; d TriNetX LLC, Cambridge, MA, USA; e Liverpool Centre for Cardiovascular Science at University of Liverpool, Liverpool John Moores University and Liverpool Heart & Chest Hospital, Liverpool, UK; f Danish Center for Health Services Research, Department of Clinical Medicine, Aalborg University, Aalborg, Denmark; g Visiting Fellow, Centre for Biomechanics and Rehabilitation Technologies, Staffordshire University, Stoke-on-Trent, UK

**Keywords:** Type 2 diabetes, Tirzepatide, Semaglutide, Obesity, Cardiovascular outcomes

## Abstract

**Background:**

Tirzepatide, a novel dual agonist of glucagon-like-peptide-1 (GLP-1) and glucose-dependent insulinotropic polypeptide (GIP), has demonstrated greater magnitude of weight loss compared to semaglutide in a phase 3 clinical trial. However, the effect of tirzepatide on incidence of type 2 diabetes (T2D) in individuals with overweight and obesity, and the effect on major adverse cardiovascular outcomes in individuals with pre-existing T2D, remains unknown.

**Methods:**

We performed a retrospective cohort study of anonymised electronic medical records using the TriNetX network (TriNetX LLC, Cambridge, MA, USA) a global federated database. The data used in this study was collected on 5th June 2024. Two cohorts of individuals were generated: **1)** without pre-existing T2D and, **2)** with T2D. We adopted an active comparator new user design on new initiations of either tirzepatide *or* semaglutide therapy. Analysis began from the index event which was defined as individuals on respective therapy for 6 months only. Analysis of outcomes was conducted off-drug, in individuals without a pre-existing history of the disease of interest. Individuals were followed up for 12 months post the index event. **Primary outcome** for **cohort 1** was incidence of T2D, and for **cohort 2** was composite: all-cause mortality, cerebral infarction, acute coronary syndrome, and heart failure. **Secondary outcomes** for **cohort 1** were change in HbA1c and body weight and for **cohort 2**: incidence of micro- and macrovascular complications, suicidal ideation and/or attempt, and all-cause mortality. We propensity score matched (1:1) for potential confounders: baseline demographics, socioeconomic circumstances, HbA1c, weight, relevant co-morbidities, and anti-obesity, hypoglycaemic and cardioprotective agents.

**Findings:**

The study population without T2D consisted of 13,846 individuals, equally split between tirzepatide and semaglutide users. Tirzepatide was associated with both lower risk for incident T2D (HR 0.73, 95% CI 0.58–0.92, p < 0.001) and greater weight loss (−7.7 kg, [95% CI −6.8, −8.5 kg], p < 0.001), compared to semaglutide (−4.8 kg, [95% CI −3.9, −5.6 kg], p < 0.001). In individuals with pre-existing T2D (n = 8446), tirzepatide was associated with lower risk of the composite outcome (HR 0.54, 95% CI 0.38–0.76, p < 0.001), cerebral infarction (HR 0.45, 95% CI 0.24–0.84, p = 0.010) and all-cause mortality (HR 0.33, 95% CI 0.15–0.73, p = 0.004) compared to semaglutide.

**Interpretation:**

Tirzepatide is associated with significantly reduced risk of developing T2D and major adverse cardiovascular events in individuals living with obesity and T2D respectively. Randomised controlled trials investigating the utility of dual incretin agonists in the primary prevention of T2D and cardiovascular disease in higher risk populations are now required.

**Funding:**

Nil.

**Research in context**

**Evidence before this study**

Studies of tirzepatide, a novel dual glucagon-like-peptide-1 (GLP-1) and glucose-dependent insulinotropic polypeptide (GIP) receptor agonist, demonstrates seemingly greater weight loss and HbA1c reduction when compared to studies of semaglutide in people with type 2 diabetes (T2D). We searched PubMed (up to 28th March 2024) for studies of the effect of tirzepatide in the primary prevention of T2D in people living with obesity, but this is unknown, and currently remains off label.

**Added value of this study**

We present a propensity-matched study investigating and comparing the use of tirzepatide vs semaglutide for the primary prevention of T2D, in a cohort of over 13,000 people at high risk for T2D. Tirzepatide was associated with greater weight loss and reduced risk of T2D compared to semaglutide and should therefore be considered in future risk reduction studies.

**Implications of all the available evidence**

Early prevention of T2D is the cornerstone of all national weight loss programmes and is a critical pillar in tackling the twin obesity and T2D pandemics. The findings of this retrospective analysis suggest that tirzepatide may be more effective than semaglutide in preventing new onset T2D in individuals living with obesity. These findings need to be confirmed in randomised, controlled studies, in high-risk individuals without pre-existing T2D.

## Introduction

Obesity is a major worldwide health concern. Current estimates of the number of adults living with obesity is at 650 million,^1^ with a further 340 million children and adolescents.^2^ Overweight and obesity represent the primary risk factors for the development of type 2 diabetes (T2D), with T2D driving the excess morbidity and mortality seen in this population.^3^ The US's Centers for Disease Control and Prevention (CDC) estimate over 97 million people aged 18 or over have prediabetes, ∼38% of the adult US population.^4^ The total direct and indirect costs of diagnosed diabetes is estimated to exceed $410 billion, with the majority of expenditure directly attributable to diabetes-related complications.^5^ Therefore, the potential economic and societal impact of pharmacological therapies that reduce the conversion from prediabetes to T2D would be huge. Effective management of obesity is multifactorial, consisting of behavioural interventions, dietary modification, increased physical activity, pharmacological therapies, metabolic and bariatric surgery.^6^ Surgical interventions offer the greatest magnitude of sustained weight loss but there are often barriers to access, a risk of anaesthetic, surgical and potentially post-operative complications.^7^

Glucagon like peptide-1 receptor agonists (GLP-1 RA), by slowing gastric emptying and reducing appetite, are an increasingly utilised pharmacotherapy in the treatment of overweight and obesity.^8^ The once weekly preparation of GLP-1 RA, semaglutide, offers greater weight loss compared to the administration of earlier generation GLP-1 RAs such as exenatide.^9^

Recently, tirzepatide, a dual agonist of GLP-1 and glucose-dependent insulinotropic polypeptide (GIP), has demonstrated greater degree of weight loss compared to single peptide (GLP-1) receptor agonists.^10^ Weight loss is unequivocally the most effective intervention to reduce the subsequent risk of T2D in individuals living with overweight or obesity, while in individuals who have already developed T2D, weight loss is associated with fewer adverse health events, lesser micro- and macrovascular complications, greater overall survival and remission.^11^ However, the clinical effect of the newly introduced dual agonist, tirzepatide, on the incidence of T2D in individuals living with overweight and obesity, and indeed the impact on cardiovascular outcomes remains unexplored. Controversy surrounding semaglutide and the purported increased risk of suicidal thoughts, initially raised by the Icelandic Medicines Agency, has been subject to extensive review and has since been disproven.^12^^,^^13^ The risk of suicidal attempt and ideation has not been studied with tirzepatide use. Hence, in this study, we took advantage of a large international federated database of electronic health records to determine and compare the relative association of tirzepatide and semaglutide on i) incident T2D in individuals living with obesity, and ii) to assess their relative effects on the rates of micro-, macrovascular complications, all-cause mortality, and suicidal attempt/ideation, in individuals with pre-existing T2D.

## Methods

### Network characteristics

We performed a retrospective cohort study using the TriNetX (TriNetX LLC, Cambridge, MA, USA) platform. The TriNetX research platform is a federated database providing access to real-time anonymised electronic medical records. TriNetX has data usage and publication agreements in place with all health care organisations (HCOs). TriNetX provides comprehensive datasets that encompass a wide range of variables, including patient demographics, diagnoses, procedures, medications, and laboratory results collected mostly from HCOs electronic medical records (EMRs). This extensive data collection ensures that we capture a holistic view of patient health and related outcomes, contributing to the completeness of our analyses. However, due to the nature of the data source, this dataset may face some typical data quality challenges of EMRs such as incomplete or inaccurate data entries, under-reporting of certain conditions, limited granularity and exclusion of data not integrated into the HCO's EMR. Nevertheless, TriNetX employs data validation processes to ensure the accuracy and reliability of its data. These processes include regular data quality checks to identify and correct discrepancies, validation against external benchmarks to ensure consistency and accuracy, and collaboration with data contributors to resolve any identified issues and improve data quality continuously. We utilised the TriNetX Global Collaborative network which comprises of >135 million individuals across over 100 health care organisations (HCOs), primarily secondary and tertiary units in North America. The data used in this study was collected on 5th June 2024.

### Cohort without pre-existing T2D (cohorts 1a and 2a)

**Fig. 1:**
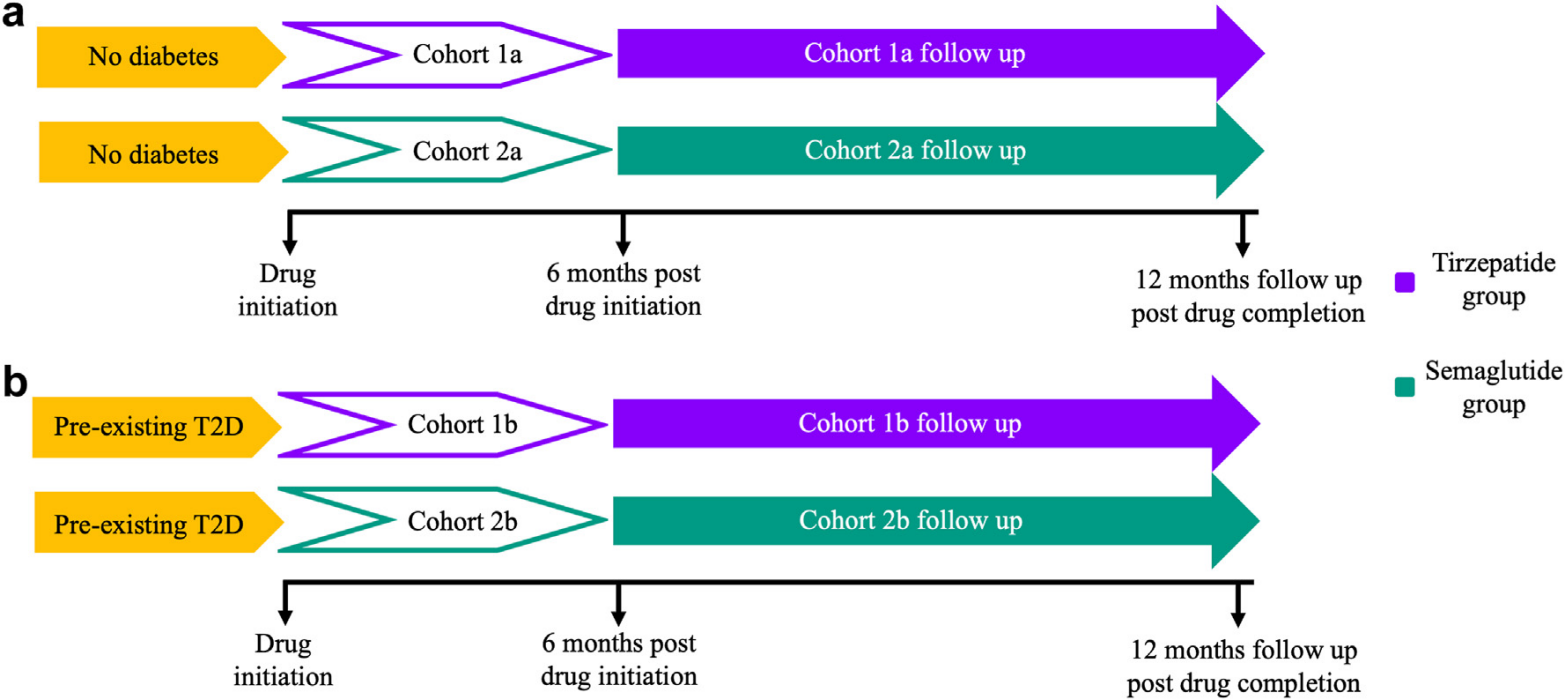
Timeline of included individuals for the cohort analysis of individuals **(a)** without T2D and time to incident T2D, **(b)** with T2D. all-cause mortality and rates of complications.

We identified all adults aged 18 or over without a pre-existing diagnosis of any diabetes mellitus. Two arms were generated for comparison. **Cohort 1a** consisted of individuals prescribed tirzepatide for 6 months only (the **tirzepatide group**). We excluded individuals who were ever prescribed any GLP-1 RA or pramlintide. **Cohort 2a** consisted of individuals prescribed semaglutide for 6 months only (the **semaglutide group**). We excluded individuals who were ever prescribed any other GLP-1 RA, tirzepatide or pramlintide. Individuals were not prescribed any incretin-based therapy beyond the 6-month stipulated period. We adopted an active comparator new user design where analysis was of new starters of each drug. Active comparators often share similar treatment indications and patient characteristics, which helps control for confounders that might otherwise skew the results, and we can better establish temporal relationships between drug exposure and outcomes, as the initiation of the drug is clearly defined.^14^ Analysis began from the index event which was defined as completion of 6 months of respective therapy. Individuals were followed up for 12 months post index event (Fig. 1a). **The primary outcome was incidence of T2D**. **Secondary outcomes were: 1) change in HbA1c and body weight and 2) incidence of clinically significant hypoglycaemia** (ICD-10 E11.64, E16.2) **and acute pancreatitis** (ICD-10 K85). Time to event analysis was conducted off-drug, tirzepatide is known to be more effective in reducing gylcaemia than semaglutide, therefore, if analysis was performed on-drug, it would simply reflect the greater glycaemic effect of tirzepatide. Cohorts were 1:1 propensity score matched (PSM) using greedy nearest neighbour matching with a caliper of 0.1 pooled standard deviations. We matched individuals for: age at index event, sex, ethnicity, HbA1c, weight, tobacco use (ICD-10 Z72.0), socioeconomic and psychosocial circumstances (ICD-10 Z55-Z65) and use of metformin, SGLT2i, orlistat, bupropion, naltrexone, topiramate, phentermine, corticosteroids, lipid lowering agents, antipsychotics, cyclosporin and tacrolimus.

### Cohort with T2D (cohorts 1b and 2b)

We additionally identified all adults aged 18 years and over with an existing diagnosis of T2D. Two further arms were generated for comparison. **Cohort 1b** consisted of individuals with T2D who were prescribed tirzepatide for 6 months only (the **T2D + tirzepatide group**) while **cohort 2b** consisted of individuals with T2D who were prescribed semaglutide for 6 months only (the **T2D + semaglutide group**). We applied the same exclusion criteria to both groups as described for cohorts 1a and 2a. Analysis began from the index event which was defined as being on respective therapy for 6 months only. Individuals were followed up for 12 months post index event (Fig. 1b). Individuals were not prescribed any incretin-based therapy beyond the 6-month stipulated period. **The primary outcome was time to first composite event: all-cause mortality, cerebral infarction** (ICD-10 I63)**, acute coronary syndrome** (ICD-10 I20.0, I21-22) **or heart failure** (ICD-10 I50)**. Secondary outcomes were: 1) all-cause mortality, 2) incidence of macrovascular complications:** ischaemic heart disease (ICD-10 I20–I25), heart failure (ICD-10 I50), cerebral infarction (ICD-10 I63)**, 3) incidence of microvascular complications:** diabetic retinopathy (ICD-10 E11.31–35), nephropathy (ICD-10 E11.1), neuropathy (ICD-10 E11.40–43) **and 4) incidence of suicidal ideation and/or attempt** (ICD-10 R45.851 & T14.91). Pre-existing diagnosis of a disease may get periodically coded for a variety of reasons and not necessarily reflect severity of disease, therefore, to better establish temporal relationships between drug exposure and new outcomes, individuals without a history of a micro or macrovascular outcome of interest were excluded from prospective analysis of that specific outcome only. Individuals with a history of any of the constituent composite events were again excluded from time to composite outcome analysis in order to better establish the primary preventative effect of tirzepatide vs semaglutide. Time to event analysis was conducted off drug. We matched individuals for: age at index event, sex, ethnicity, proportion of individuals with a HbA1c (≥6.5% and ≤7.5%; >7.5% and ≤8.5%; >8.5% and ≤9.5% and >9.5%), weight, estimated glomerular filtration rate (eGFR), triglycerides, total cholesterol, systolic blood pressure, tobacco use (ICD-10 Z72.0), socioeconomic and psychosocial circumstances (ICD-10 Z55-Z65), ischaemic heart diseases (ICD-10 I20-I25), cerebrovascular diseases (ICD-10 I60-I69), hypertensive diseases (ICD-10 I10-11A), heart failure (ICD-10 I50), atrial fibrillation and flutter (ICD-10 I48), chronic kidney disease (CKD) (ICD-10 N18), history of any neoplasms (ICD-10 C00-D49), chronic lower respiratory diseases (ICD-10 J40-J4A) and use of all oral hypoglycaemic agents, insulin, lipid lowering agents, angiotensin converting enzyme inhibitors, angiotensin II receptor antagonists, diuretics, beta blockers, anti-arrhythmics, platelet aggregation inhibitors and anticoagulants.

### Statistics

Statistical analysis is conducted within the TriNetX platform using the R survival package. Baseline characteristics are presented as mean with standard deviation (SD). PSM was performed using logistic regression. TriNetX uses ‘greedy nearest-neighbour matching’ with a caliper of 0.1 pooled standard deviations and difference between propensity scores <0.1. We assessed covariate balance between groups using the strictly standardised mean difference (SSMD). Variables with strictly SSMD <0.1 were considered well matched. Thereafter, univariate survival analysis was conducted in-situ within TriNetX to estimate the probability of the outcome of interest at daily time intervals with censoring applied. When the last fact (outcomes of interest, date of death, end of data collection, or loss to follow up) in the patient's record was in the time window for analysis, the patient was censored on the day after the last fact in their record. Hazard ratios (HR) and 95% confidence intervals (CI) were used to describe the relative hazard of the outcomes based on a comparison of time-to-event rates. A Log Rank test with p values, and Kaplan Meier survival curves, were also generated. Additionally, for sensitivity analysis, we calculated the E-Value for each outcome of interest. E-value is defined as the minimum strength of association, on the risk ratio scale that an unmeasured confounder would need to have with both treatment and outcome to fully explain away a specific treatment–outcome association.^15^ Statistical significance was set at the 5% level.

### Ethics

Data collection, processing, and transmission are performed in compliance with all Data Protection laws applicable to the contributing HCOs, including the EU Data Protection Law Regulation 2016/679, the General Data Protection Regulation on the protection of natural persons regarding the processing of personal data, and the Health Insurance Portability and Accountability Act, the US federal law which protects the privacy and security of healthcare data. The TriNetX Global Collaborative Network is a distributed network (with most HCOs located in the USA), and analytics are performed at the HCO with only aggregate results being surfaced and returned to the platform. Data usage and publication agreements are in place with all HCOs. TriNetX, LLC is compliant with the Health Insurance Portability and Accountability Act (HIPAA), the US federal law which protects the privacy and security of healthcare data, and any additional data privacy regulations applicable to the contributing HCO. TriNetX is certified to the ISO 27001:2013 standard and maintains an Information Security Management System (ISMS) to ensure the protection of the healthcare data it has access to and to meet the requirements of the HIPAA Security Rule. Any data displayed on the TriNetX Platform in aggregate form, or any patient level data provided in a data set generated by the TriNetX Platform, only contains de-identified data as per the de-identification standard defined in Section 164.514(a) of the HIPAA Privacy Rule. The process by which the data is de-identified is attested to through a formal determination by a qualified expert as defined in Section 164.514(b) (1) of the HIPAA Privacy Rule. Because this study used only de-identified patient records and did not involve the collection, use, or transmittal of individually identifiable data, this study was exempted from Institutional Review Board approval.

### Role of funding source

This study did not receive any funding.

## Results

### Cohort without pre-existing T2D

**Fig. 2:**
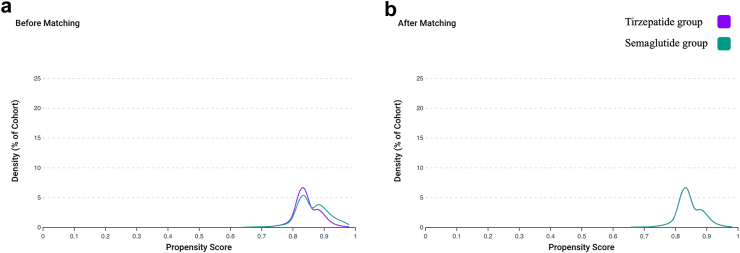
Propensity score density function for cohorts 1a and 2a.

We identified a total of 53,386 individuals. 6928 (13%) were prescribed tirzepatide and 46,458 (87%) prescribed semaglutide. Post PSM, there were 6923 in each group. Mean age was 47.5 ± 11.8 years and 47.5 ± 11.9 years for the for the tirzepatide and semaglutide groups respectively, 73% were female and most individuals were white (Table 1). Both groups were well matched for baseline HbA1c and body weight (Fig. 2). Mean follow up for the tirzepatide group was 302 days and 312 days for the semaglutide group.

**Table 1:** Baseline patient demographics and characteristics post PSM.

	Tirzepatide group (n = 6923)	Semaglutide group (n = 6923)	SSMD
**Demographics**			
Age at index event (years)	47.5 ± 11.8	47.5 ± 11.9	0.002
Sex (Female) [%]	73	73	0.006
Race (White/Black/Asian) [%]	73/11/1	74/10/1	0.010/0.004/0.004
**Biochemistry and anthropometric measures**			
Weight (kg)	99.9 ± 25.8	101.4 ± 24.3	0.061
HbA1c (DCCT %)	5.4 ± 0.7	5.5 ± 0.7	0.064
**Drug treatment/use [%]**			
Metformin	0.8	0.8	0.003
SGLT2 inhibitors			
Empagliflozin	0.1	0.1	<0.001
Dapagliflozin	0.1	0.1	<0.001
Canagliflozin	0	0.1	0.054
Ertugliflozin	0	0	N/A
Anti-obesity medication			
Orlistat	0	0.1	0.054
Bupropion	1.4	1.4	0.001
Naltrexone	0.1	0.2	0.004
Topiramate	0.3	0.3	0.005
Phentermine	0.7	0.7	0.004
Other			
Adrenal corticosteroids	22.3	22.6	0.008
Lipid lowering agents	10.2	10.6	0.016
Antipsychotics	2.2	1.8	0.027
Cyclosporin	0.3	0.3	<0.001
Tacrolimus	0.3	0.3	<0.001
**Lifestyle factors [%]**			
Tobacco use	0.2	0.1	0.010
Persons with potential health hazards related to socioeconomic and psychosocial circumstances	0.2	0.2	0.011

SSMD: strictly standardised mean difference.

**Fig. 3:**
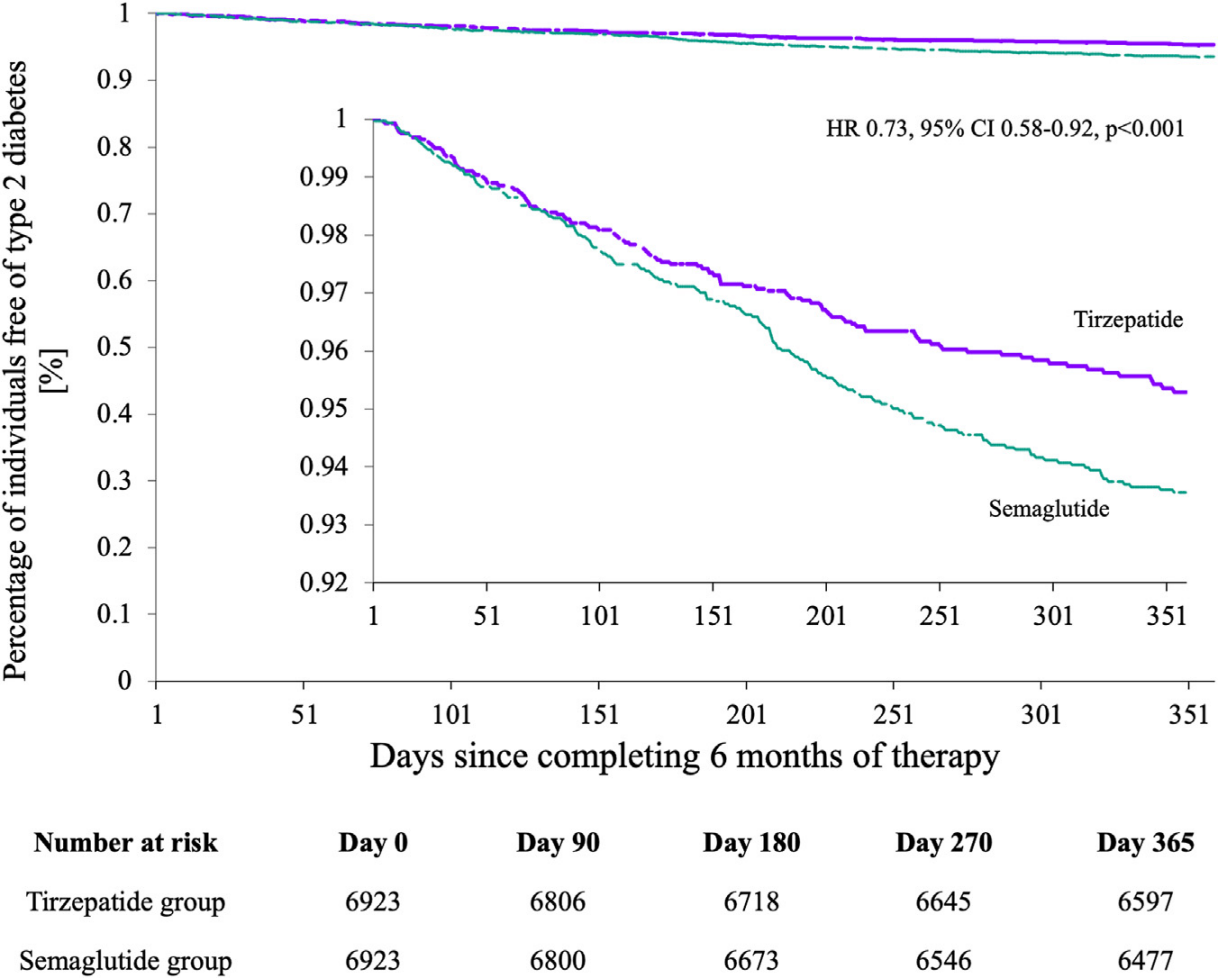
Kaplan–Meier estimates of event free rate between tirzepatide and semaglutide groups.

The tirzepatide group was associated with a reduced risk of developing T2D over 1 year, which increased with time (hazard ratio (HR) 0.73, 95% CI 0.58–0.92, p < 0.001, E value = 1.79) (Fig. 3).

Weight loss at 1 year was greater in the tirzepatide group (−7.7 kg [95% CI −6.8, −8.5 kg], p < 0.001) than the semaglutide group (−4.8 kg [95% CI −3.9, −5.6 kg], p < 0.001). Reduction in HbA1c was greater in the tirzepatide (−0.24% [95% CI −0.22, −0.26%], p < 0.001) than semaglutide (−0.1% [95% CI −0.13, −0.07%], p < 0.001) group.

There was no difference in rates of clinically significant hypoglycaemia (HR 1.07, 95% CI 0.62–1.86, p = 0.804) or acute pancreatitis (HR 0.98, 95% CI 0.49–1.96, p = 0.194).

### Cohort with T2D

**Fig. 4:**
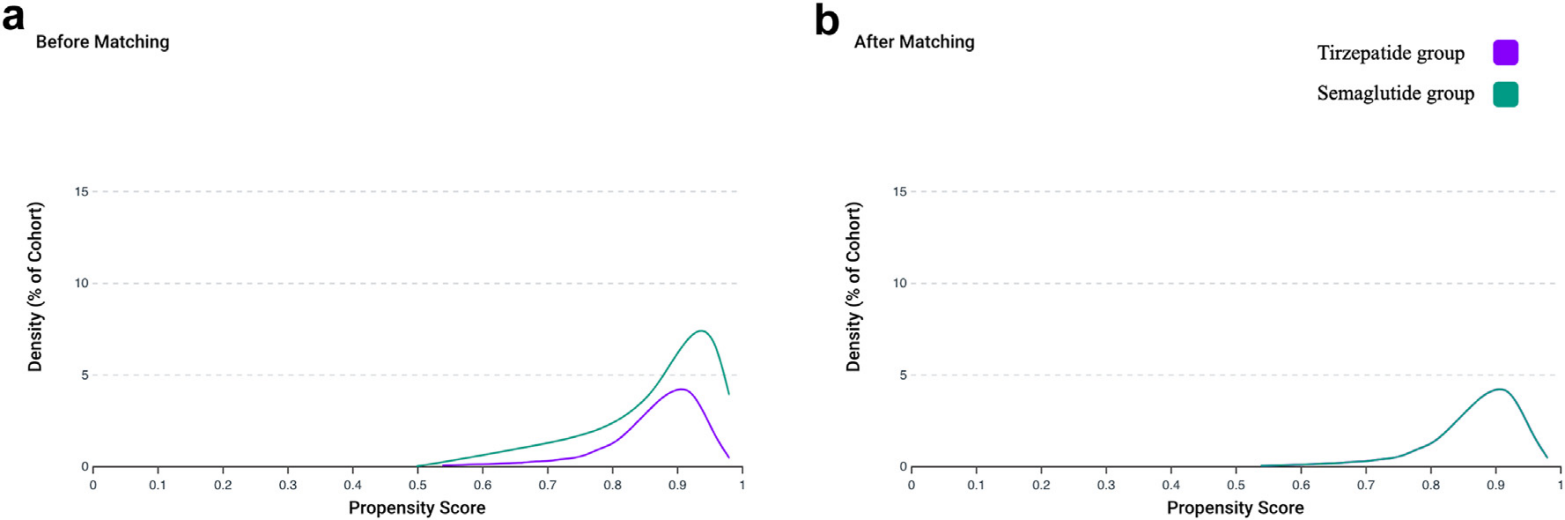
Propensity score density function for cohorts 1b and 2b.

We identified a total of 50,456 individuals with T2D who satisfied our inclusion criteria. 4225 (8.4%) were prescribed tirzepatide and 46,231 (91.6%) semaglutide. Post PSM, there were 4223 in each group. Mean age was 53.9 ± 10.7 years and 54.0 ± 12.2 years, 60% were female (Table 2). Body weight and proportion of individuals per HbA1c strata were well balanced (Fig. 4). Mean follow up for the tirzepatide group was 328 days and 339 days for the semaglutide group.

**Table 2:** Baseline patient demographics and characteristics for the T2D groups, post PSM.

	Tirzepatide group (n = 4223)	Semaglutide group (n = 4223)	SSMD
**Demographics**			
Age at index event (years)	53.9 ± 10.7	54.0 ± 12.2	0.012
Sex (Female) [%]	60	60	0.002
Race (White/Black/Asian) [%]	68/15/3	69/14/3	0.027/0.005/0.010
**Biochemistry and anthropometrics**			
Weight (kg)	107.1 ± 26.6	105.1 ± 25.9	0.080
Estimated glomerular filtration rate (ml/min/1.73 m^2^)	83.8 ± 23.4	82.9 ± 26.0	0.035
Triglycerides (mmol/mol)	1.9 ± 1.4	2.0 ± 1.4	0.071
Total cholesterol (mmol/mol)	4.2 ± 1.1	4.2 ± 1.1	0.055
Systolic blood pressure (mmHg)	128.5 ± 16.4	128.9 ± 16.1	0.027
HbA1c [%]			
≥6.5%, ≤7.5%	40.9	39.8	0.007
>7.5%, ≤8.5%	27.9	27.5	0.001
>8.5%, ≤9.5%	15.9	17.1	0.031
>9.5%	15.3	15.6	0.010
**Co-morbidities [%]**			
Ischaemic heart diseases	10.5	10.8	0.008
Cerebrovascular diseases	2.4	2.2	0.011
Hypertensive diseases	71.0	71.3	0.005
Heart failure	5.2	5.5	0.013
Atrial fibrillation & flutter	4.1	4.1	0.002
Chronic kidney disease	9.3	9.4	0.001
Neoplasms	18.0	18.1	0.004
Chronic lower respiratory diseases	14.6	14.4	0.005
**Drug treatment [%]**			
Oral hypoglycaemic agents	63.9	64.6	0.015
Insulin	35.4	35.8	0.010
Lipid lowering agents	56.0	56.0	0.001
ACE inhibitors	23.7	24.4	0.016
Diuretics	30.9	30.5	0.007
Beta blockers	23.7	23.1	0.015
Angiotensin II inhibitors	25.2	24.7	0.011
Antiarrhythmics	26.0	25.2	0.018
Platelet aggregation inhibitors	10.4	10.7	0.009
Anticoagulants	10.5	9.8	0.025
**Lifestyle factors [%]**			
Tobacco use	2.0	2.2	0.016
Persons with potential health hazards related to socioeconomic and psychosocial circumstances	1.8	1.5	0.019

SSMD: strictly standardised mean difference.

Tirzepatide was associated with reduction in the composite outcome of all-cause mortality, acute coronary syndrome, cerebral infarction and heart failure (HR 0.54, 95% CI 0.38–0.76, p < 0.001, E value = 2.43) (Table 3).

**Table 3:** Clinical outcomes of the T2D cohort.

	Sample size	Outcome (n)	HR (95% confidence interval)	p value	*E value*
**All-cause mortality**					
Tirzepatide	4223	8	**0.33 (0.15–0.73)**	**0.004**	**3.69**
Semaglutide	4223	25			
**Macrovascular complications**					
**Ischaemic heart disease**					
Tirzepatide	3503	96	0.99 (0.75–1.31)	0.923	1.00
Semaglutide	3467	99			
**Cerebral infarction**					
Tirzepatide	4108	14	**0.45 (0.24–0.84)**	**0.010**	**2.86**
Semaglutide	4113	32			
**Heart failure**					
Tirzepatide	3892	32	0.71 (0.45–1.12)	0.140	1.00
Semaglutide	3869	46			
**Acute coronary syndrome**					
Tirzepatide	4025	55	0.96 (0.67–1.39)	0.840	1.00
Semaglutide	4041	59			
**Composite (heart failure, acute coronary syndrome, cerebral infarction, all-cause mortality)**^a^					
Tirzepatide	3668	50	**0.54 (0.38–0.76)**	**<0.001**	**2.43**
Semaglutide	3623	94			
**Microvascular complications**					
**Diabetic retinopathy**					
Tirzepatide	3853	239	1.12 (0.93–1.35)	0.225	1.00
Semaglutide	3867	216			
**Diabetic nephropathy**					
Tirzepatide	3925	157	1.18 (0.94–1.49)	0.151	1.00
Semaglutide	3969	135			
**Diabetic neuropathy**					
Tirzepatide	3314	592	**1.14 (1.02–1.29)**	**0.024**	**1.53**
Semaglutide	3379	528			
**Other complications**					
**Suicidal attempt and ideation**					
Tirzepatide	4223	7	0.55 (0.22–1.37)	0.192	1.00
Semaglutide	4223	13			

Bold indicates statistical signifcance.

a Individuals were censored at the first coding of a constituent composite outcome. The total number of individuals experiencing the composite outcome were less than the sum of the individual events because to better ascertain the primary preventative effect of tirzepatide on time to first major adverse cardiovascular event (MACE), individuals with a history of any of the constituent events were excluded from analysis of the composite outcome.

Treatment with tirzepatide was associated with significant reduction in all-cause mortality over 12 months compared to semaglutide (HR 0.33, 95% CI 0.15–0.73, p = 0.004, E value = 3.69) (Table 3).

Tirzepatide use was associated with reduction in cerebral infarction (HR 0.45, 95% CI 0.24–0.84, p = 0.010, E value = 2.86) but not heart failure (HR 0.71, 95% CI 0.45–1.12, p = 0.140) or ischaemic heart disease (HR 0.99, 95% CI 0.75–1.31, p = 0.923) (Table 3).

Tirzepatide was associated with an increase in incident diabetic neuropathy (HR 1.14, 95% CI 1.02–1.29, p = 0.024, E-value = 1.53) but not retinopathy (HR 1.12, 95% CI 0.93–1.35, p = 0.225) or nephropathy (HR 1.18, 95% CI 0.94–1.49, p = 0.151) (Table 3).

There was no difference in risk of suicide attempt and ideation between groups (HR 0.55, 95% CI 0.22–1.37, p = 0.192) (Table 3).

## Discussion

We have, to the best of our knowledge, performed the first real world study to demonstrate tirzepatide, a novel GLP-1/GIP dual receptor agonist, compared to semaglutide, the most potent GLP-1 RA, is associated with a 27% lower rate of incident T2D over a 1 year follow up. Additionally, in individuals with T2D, tirzepatide was associated with a 46% reduction of risk of first major adverse cardiovascular event (MACE), a 55% reduced risk of cerebral infarction, and a two third reduction in all-cause mortality. Given this real world, retrospective data, with the inherent limitations, our findings may be considered as hypothesis-generating, with head-to-head randomised controlled trials required to provide confirmation of superiority (or non-inferiority).

To date, there is limited evidence around incident T2D following treatment with tirzepatide. The SURMOUNT programme established the impact of tirzepatide on weight loss in patients with obesity, however, did not report incident T2D, whilst the SURPASS programme assessed the impact of tirzepatide on glycaemic control in patients with established T2D.^16^^,^^17^ To address these limitations, we explored the impact of tirzepatide, compared with semaglutide, on the incidence of T2D, in a large secondary care cohort.

Similar to SURMOUNT-1, our cohort included people primarily living with obesity, and as expected, tirzepatide had a greater magnitude of weight loss than semaglutide.^18^ Weight loss may contribute towards the reduced risk of incident T2D in our tirzepatide arm, as there exists a progressive dose–response relationship between weight loss and incident T2D.^19^ For every kilogram of weight loss, a 16% reduction in risk in incident T2D is observed, adjusted for changes in diet and activity. Despite this, the magnitude of weight loss in our tirzepatide cohort was lower than previously described.^18^ Conversely, the magnitude of effect size seen following tirzepatide treatment is large, reducing the risk of incident T2D by 27%, which is conservative given our control group consisted of patients prescribed semaglutide; a potent GLP-1 RA.^20^ For comparison, the UK diabetes prevention programme delivered an estimated reduced risk of incident T2D of 6.2–7.3%^21^ with a mean weight loss of participants completing the programme of 4.76 kg (4.60–4.92 kg),^22^ considerably lower than our study demonstrate with tirzepatide. Lower than expected weight loss compared to RCTs may be explained by the real-world nature of our data. Additionally, we cannot accurately comment on the drug dose prescribed, nor the number of doses each participant received; it is possible that weight regain occurs following treatment cessation with both GLP-1 RAs, and GLP-1/GIP dual agonists.^23^

Magnitude of HbA1c reduction was less than reported from the SURPASS programme. A key distinction to be made is that change in HbA1c with tirzepatide use, to date, has not been evaluated in a population living with obesity but without T2D. The magnitude of HbA1c reduction with GLP-1 use is greater with a higher starting baseline HbA1c. The SELECT trial, evaluating semaglutide in obesity without diabetes, reported a modest −0.31% reduction in HbA1c,^24^ similar to our findings. The effect of GLP-1 and GLP-1/GIP dual agonists in people with relative normoglycemia is unknown but may demonstrate a similar attenuation as semaglutide.

The SURPASS program included participants across the severity spectrum of existing T2D diagnosis: from patients treated with lifestyle intervention, to individuals requiring insulin.^17^^,^^25^ In SURPASS, participants prescribed tirzepatide had greater weight loss, as well as reductions in HbA1c, blood pressure, and improved lipid profiles, compared to controls or placebo, all of which may contribute to the improved composite cardiovascular outcome seen in our tirzepatide cohort.^26^, ^27^, ^28^

Meta-analysis of SURPASS trials did not demonstrate a reduced risk of MACE, or all-cause mortality, however all individual endpoints analysed in this meta-analysis had a HR < 1.0, with a pooled sample size around half of our current study. We therefore suggest that this meta-analysis may have been underpowered to detect this important risk reduction.^29^ However, this meta-analysis demonstrated greater cardiovascular protection in patients with higher cardiovascular risk at the initiation of tirzepatide treatment,^30^ consistent with our study findings whereby the burden of comorbidities was high in our tirzepatide cohort. Conversely, a second, more recent, meta-analysis, demonstrated reduced MACE in patients treated with tirzepatide, and additionally identified beneficial cardiac effects through reduced cardiomyocyte death, fibrosis, and hypertrophy in the presence of hyperglycaemia.^31^ Our data suggests that this reduced risk of MACE is largely driven by a significant reduction in the rates of cerebral infarction. Although the meta-analysis by *Taktaz* et al. did not present data for individual endpoints, a 5-year modelling study suggests that tirzepatide reduces MACE over semaglutide and insulin treatment, with the greatest contributor being reduction in the incidence of cerebral vascular accident.^32^ Additionally, *Shu Niu* et al. demonstrated a reduced risk of composite microvascular disease in patients treated with tirzepatide, without providing distinction between individual endpoints.^33^

**Fig. 5:**
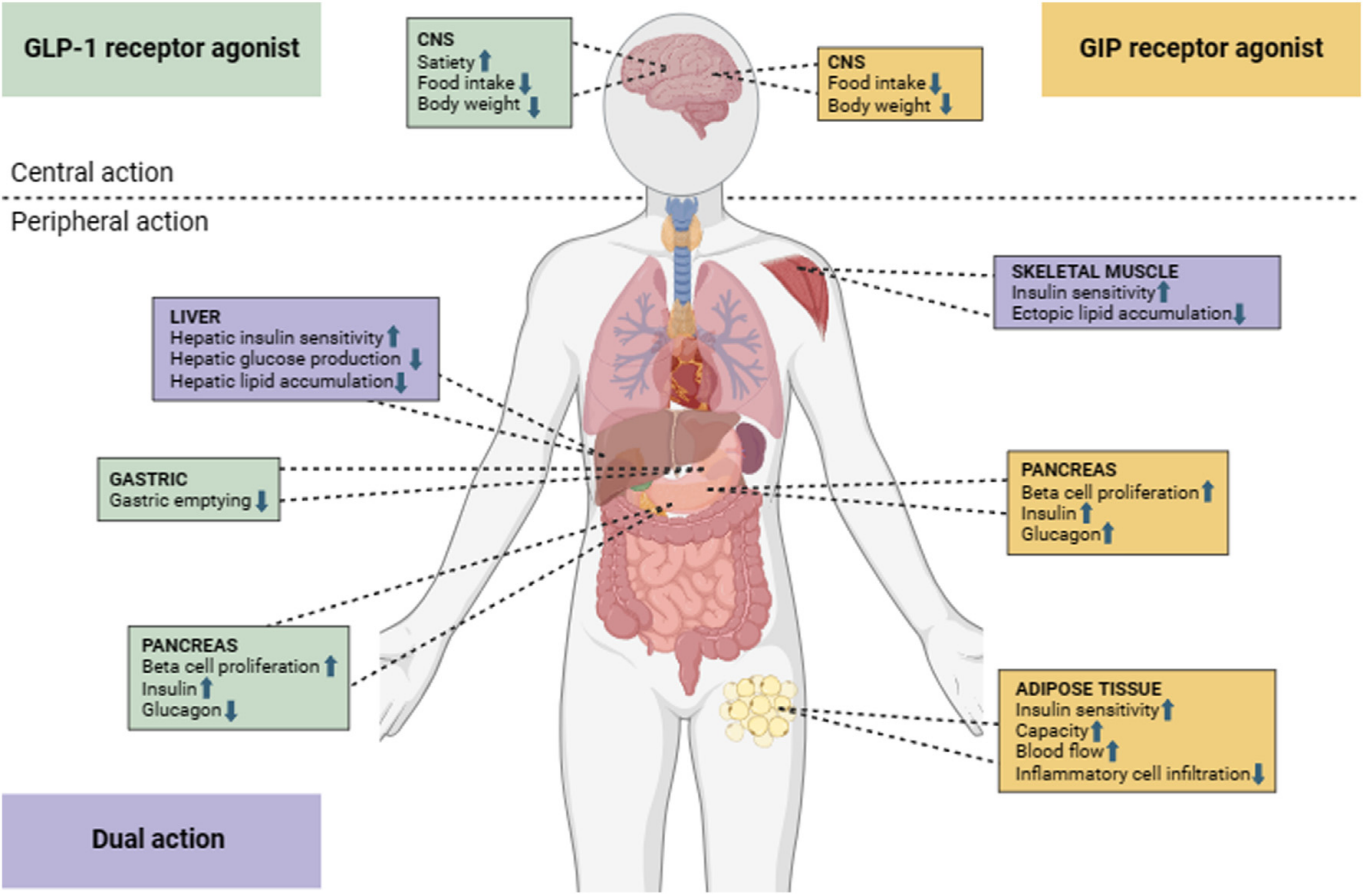
Comparison of metabolic targets of GLP-1 RA and GIP RA. The adipocytes located in the gluteofemoral region reflect subcutaneous adipose tissue globally.

We therefore suggest that other mechanisms, beyond weight loss, may contribute towards a lower T2D incidence, and MACE, in patients treated with tirzepatide. Although our study did not directly examine mechanistic pathways, animal models suggest it is biologically plausible that dual agonism of both GLP-1 and GIP receptors, following tirzepatide treatment,^34^ enhances their individual incretin response, in amplification of insulin secretion beyond that obtained by activation of either axis in isolation; either by concomitant, or sequential, activation of the two hormone receptors^35^ (Fig. 5). Therefore, the structure of tirzepatide may provide superiority over a GLP-1 analogue such as semaglutide. Moreover, improvement in cardiovascular risk factors, including lipid profile and carotid-intima media thickness, with potent GLP-1 RA treatment is well established,^36^ and may contribute to the reduced risk of MACE, and specifically cerebral infarction, established in our study.^37^

Tirzepatide was associated with an increase in incidence of diabetic neuropathy, and while the raw numbers of new diabetic retinopathy and nephropathy were greater with tirzepatide use, this did not reach statistical significance. Significant and rapid reduction of HbA1c is associated with new or worsening of existing neuropathy, a term coined treatment induced neuropathy, the pathophysiology of which remains poorly understood.^38^

Finally, it is imperative to address concerns over reports of suicidal ideation associated with GLP-1 RA treatment. A recent TriNetX analysis concluded that patients living with overweight or obesity, who were treated with semaglutide, compared to non-GLP-1 RA anti-obesity medications, had a lower risk of suicidal ideation; with the findings replicated in patients with T2D.^13^ Reassuringly, our findings suggest no significant statistical difference in suicidal attempt or ideation between participants treated with tirzepatide or semaglutide.

We must acknowledge limitations to our work. Firstly, these are real-world data, and comparisons are not randomised, nor controlled, as evidenced by the inferior weight loss demonstrated compared to clinical trials. Second, resulting from data being extracted from electronic health records of an administrative database, there is potential for a lack of data completeness. For example, data may not be recorded by the HCO, or other data recorded in free text which we are unable to extrapolate. Information concerning dosage, and rate of dose escalation of tirzepatide and semaglutide were not available to us, and we were unable to comment on the dose-dependent relationship of incretin-based therapies on body weight and/or level of glycaemia. Our findings are additionally limited by the short duration of follow up, primarily due to the limited availability of tirzepatide on the market. We were unable to provide possible explanations for the beneficial cardiovascular outcomes associated with tirzepatide use (such as with serial lipids and triglyceride measurements due to the infrequency that these were measurements were made). Data on minor gastrointestinal side effects that do not result in significant medical intervention would not be coded for and picked up by the network. Tirzepatide use at the time of analysis does not currently have a label in people without diabetes and hence, all use was off label. In addition, should participants move between HCO, it is possible that some of their data may not be available to us as one or more of their HCOs may not form part of the global collaborative network. Data on cardiovascular outcomes in T2D were investigated as a primary prevention measure in individuals without a pre-existing diagnosis and cannot be extrapolated to other populations. Moreover, residual bias confounding may remain possible, as with any large database study, although we attempted to reduce this through calculation of E-values as a quantitative bias analysis to assist readers in the interpretation of the strength of our results.^15^ Our active comparator new user design offers results that are more directly applicable to clinical practice because the comparator (semaglutide), is an alternative treatment option, enhancing the generalizability of our findings to broader patient populations.

In summary, from our real-world data study, compared to Semaglutide, Tirzepatide is associated with significantly reduced risk of developing T2D and major adverse cardiovascular events in individuals living with obesity and T2D respectively. Randomised controlled trials investigating the utility of dual incretin agonists in the primary prevention of T2D and cardiovascular disease in higher risk populations are now required.

## Contributors

MA, DJC and UA conceived the idea of this work. MA conducted the analysis and led the write up the original draft manuscript. AEH and NB assisted with write up of the paper. GHI facilitated access to the TriNetX platform and assisted in generating the results and analysis. SSZ, JPHW, DJC and UA provided senior author input, review and editing of the manuscript. DJC and UA oversaw all aspects of study development, design and provided senior review of the work. MA and UA verified the underlying data output from TriNetX. All authors read and approved the final version of the manuscript.

## Data sharing statement

The data that support the findings of this study are available from TriNetX, LLC but third-party restrictions apply to the availability of these data. The data were used under license for this study with restrictions that do not allow for the data to be redistributed or made publicly available. However, for accredited researchers, the TriNetX data is available for licensing at TriNetX, LLC. To gain access to the data in the TriNetX research network, a request can be made to TriNetX (https://live.trinetx.com), but costs may be incurred, a data sharing agreement would be necessary, and no patient identifiable information can be obtained. No data from Liverpool University Hospitals NHS Foundation Trust was utilized in this analysis.

## Declaration of interests

MA receives a fellowship from the Novo Nordisk UK research foundation and JDRF. DJC has received investigator-initiated grants from Astra Zeneca and Novo Nordisk, support for education from Perspectum with any financial remuneration from pharmaceutical company consultation made to the University of Liverpool. GHI is an employee of TriNetX LLC. UA has received honoraria from Eli Lilly, Procter & Gamble, Viatris, Grunenthal and Sanofi for educational meetings and funding for attendance to an educational meeting from Diiachi Sankyo. UA has also received investigator-led funding by Procter & Gamble and is a council member of the Royal Society of Medicine's Vascular, Lipid & Metabolic Medicine Section. JPHW consults widely for pharmaceutical companies in relation to obesity and diabetes (fees paid to the University of Liverpool via his institution) and has received research grants from industry. He has received lecture fees from commercial organisations including the pharmaceutical industry while providing unpaid support to various charities in relation to his interest in obesity and diabetes. All other authors declare that there are no financial relationships or activities that might bias, or be perceived to bias, their contribution to this manuscript.
